# Essential Questions on Suicide Bereavement and Postvention

**DOI:** 10.3390/ijerph9010024

**Published:** 2011-12-27

**Authors:** Karl Andriessen, Karolina Krysinska

**Affiliations:** 1 Suicide Prevention Program of the Flemish Community Mental Health Centres, 9000 Gent, Belgium; 2 Unit for Clinical Psychology Research, Faculty of Psychology and Educational Sciences, Catholic University Leuven, 3000 Leuven, Belgium; Email: karolina.krysinska@ppw.kuleuven.be

**Keywords:** suicide, prevention, postvention, bereavement, public health, policy

## Abstract

During the past decades public and research interest in postvention, *i.e.*, support for families and communities after a suicide, has increased. However, the postvention field is still facing a number of important challenges and questions. This article aims to discuss a series of essential issues on suicide bereavement and postvention, regarding the current state of the art and future developments. Who is a suicide survivor and how many suicide survivors are there? Is suicide bereavement different from other types of bereavement? What are the needs of suicide survivors and what is postvention from a clinical perspective and from a public health perspective? Can postvention be prevention? With this last question, the article concludes with a series of recommendations in order to strengthen the potential of postvention as prevention.

## 1. Introduction

Any discussion of suicide, a serious public health problem claiming globally approximately one million victims per year, will be incomplete without taking into consideration the perspective of the bereaved, or in other word, the suicide survivors. Their number is significantly higher than the number of suicide victims and some of the survivors have to cope with serious and long lasting psycho-social sequelae of the loss, including increased risk of suicidality. Although a wide range of support initiatives organised by survivors themselves, mental health professionals and concerned communities are available, numerous challenges lie ahead with regards to program and policy development, research and clinical practice, to ensure effective care for the bereaved by suicide, *i.e.*, postvention. Two basic, but nonetheless challenging questions have to be answered before starting a discussion on the potential impact of loss by suicide, the needs of the bereaved and the prerequisites of effective postvention. The first question is: how can we define a “suicide survivor”? The second question addresses the magnitude of the problem: how many suicide survivors are there?

## 2. Who Is a Suicide Survivor and How Many Survivors Are There?

There is a lack of consensus in the literature regarding the definition of a “suicide survivor”; however, the proposed definitions share several commonalities. They focus on the fact that there existed a relationship between the deceased and the bereaved, on the closeness of this relationship and/or the impact of the loss on the bereaved. For example, McIntosh [1] defined suicide survivors as “the family members and friends who experience the suicide of a loved one” (p. 146) and Andriessen [2] defined a survivor as “a person who has lost a significant other (or a loved one) by suicide, and whose life is changed because of the loss” (p. 43). Jordan and McIntosh [3] in their definition acknowledged the wide range of experiences of the bereaved: “a suicide survivor is someone who experiences a high level of self-perceived psychological, physical, and/or social distress for a considerable length of time after exposure to the suicide of another person” (p. 7). There is a distinction between “suicide survivorship” and “exposure to suicide” [2,3,4,5]. The former applies to the bereaved who had a personal and close relationship with the deceased (e.g., a friend or a family member), the latter reflects a situation of a person who did not know the deceased personally but who knows about the death through reports of others or media reports (e.g., suicide of a celebrity) or who has personally witnessed the death of a stranger (e.g., train drivers or police). 

Indeed, a suicide death can affect people in various types of relationships with the deceased: from close family members to more distant relatives, friends, neighbours, and employers [6] and several attempts at assessing the number of suicide survivors have been made. For example, Shneidman [7] has suggested that an average of six survivors are bereaved by a suicide and according to Wrobleski [8], there were 10 survivors left after a suicide death. The first systematic estimation of the number of suicide survivors, a survey among members of suicide survivor support groups [4], found that the number varied considerably depending on the type of the relationship, the frequency of contact between the deceased and the bereaved, and the age of the deceased. For example, parents who lost a child by suicide estimated that the death has left 80 suicide survivors behind, the spouses and/or partners of the suicides estimated the number at 60, while siblings and/or friends at 45–50. Five survivors after one suicide, the estimate of survivors limited to the members of a typical nuclear family, was almost identical to the original “*guesstimate”* of Shneidman [7]. 

The metaphor of a stone thrown into a lake reflects well the wide-reaching impact of suicide. It causes many ripples which turbulently affect the water’s surface. The big challenge for effective postvention is ensuring that every survivor, from the close family members and friends to those indirectly exposed to suicide, can receive help and support they need. Provision of timely and adequate services for the bereaved requires also a good understanding of the bereavement process and the needs of the survivors as a group as well as acknowledging the individual differences between the bereaved. 

## 3. Is Suicide Bereavement Different from Other Types of Bereavement?

There seems to be a gap between personal accounts of individual survivors, for example first person accounts written by survivors themselves, e.g., [9] and narratives of clinicians working with the bereaved, and outcomes of research studies [10]. In the words of Jordan [11], “there is an apparent contradiction between the perceptions of people who are bereaved by suicide and the clinicians who work closely with them, and researchers who study survivors from a greater distance with the tools of social science” (p. 92). The former indicate the uniqueness of bereavement after suicide. They focus on the experience of guilt and shame, social stigma and isolation, as well as the desperate search for the meaning of the death by the bereaved and his or her increased risk of suicide, e.g., [12]. The latter often find more similarities than differences among different groups of the bereaved in regards to major themes, the trajectory and duration of bereavement, e.g., [11,13,14]. The existing contradiction is perpetuated by the (self-)selection bias between the clinical groups and groups included in the studies (e.g., people seeking professional help might experience more so-called prolonged or complicated grief than individuals participating in bereavement surveys) as well as methodological problems of many studies, including small sample sizes, lack of control groups and lack of follow-up of the bereaved over longer periods of time [10,15]. 

In an attempt to explain the differences observed in clinical practice and research studies, Jordan & McIntosh [14] have proposed a framework encompassing various levels of grief reactions. According to this framework, in suicide bereavement one can recognize reactions present in bereavement after all types of death, such as sorrow and yearning to be reunited with the deceased, reactions characteristic for bereavement after unexpected deaths, e.g., shock and sense of unreality about the death, and elements of bereavement after violent deaths, e.g., trauma of finding a mutilated body and shattered illusion of personal invulnerability. In addition to these shared reactions, suicide survivors experience features which seem unique to suicide bereavement, such as anger at the deceased for “choosing” death over life and the feeling of abandonment. There is also accumulating clinical and empirical evidence pointing to the existence of subgroups of suicide survivors. For example, it has been reported [15] that survivors’ reactions differ as a consequence of previous history of suicidality of the deceased and the expectation of death. Those on a long-term “suicide watch” might experience after the suicide the feeling of relief (often subjectively perceived as unacceptable and coupled with guilt), while those for whom the death came unexpectedly might react with a shock, accompanied by numbness and disbelief.

## 4. What Is “Postvention” and What Are the Needs of Suicide Survivors?

Postvention can be defined as “activities developed by, with or for suicide survivors, in order to facilitate recovery after suicide and to prevent adverse outcomes including suicidal behaviour” [2], (p. 43). As such, postvention strategies aim to tackle the needs of the bereaved, and can be operationalized from two complementary perspectives: the clinical perspective, *i.e.*, the perspective of mental health professionals and services, and the public health perspective of policy development and general population strategies.

### 4.1. Postvention from the Clinical Perspective

Jordan, Feigelman, McMenamy and Mitchell [16] noted that many mental health treatments are created “top-down” by (well meaning) clinicians and researchers and suggested inclusion of the “bottom-up” approach which takes into account the “hard-won wisdom” of people bereaved by suicide (p. 116). Indeed, listening to survivors themself and exploring their needs and experiences, as well as engaging them as active partners in research [17], should be the first step in establishing effective services. Certain focus areas, such as management of the risk of psychopathology, seem the obvious goals for postvention. For example, Brent *et al*. [18] showed that children of parents who died by suicide (or in an accident) had higher rates of current and incident depression up to 21 months after the death than the non-bereaved and children of parents who died by sudden natural death. The children bereaved by suicide had also a higher rate of alcohol or substance abuse. Dyregrov and Dyregrov [19] reported that siblings of suicide victims, particularly adolescents, experienced depression, anxiety, post-traumatic and grief reactions. However, the aftermath of suicide is not limited to mental health problems. A wide variety of psycho-social needs of suicide survivors which should be met by postvention programs have been identified. These include difficulties related to the disruption of family relations and routines, functional impairments in daily activities, difficulties with social and familial relationships, spiritual struggles as well as financial and juridical problems [10,20,21,22,23]. In addition, the mechanisms of identification with the deceased, social modelling, punishment for perceived self-blame as well as genetic factors might be accountable for the increased risk of suicidal ideation and behaviour, and at-risk behaviours observed among some of the survivors [24]. Such increased risk of suicidality has been observed in both adolescent [25] and adult samples of survivors [5]. 

Suicide survivor support groups and psychotherapy seem to be promising forms of help addressing the wide variety of problems and needs of the survivors. The former may be helpful for suicide survivors in general, while the latter might be helpful especially for survivors who develop psychological and/or psychiatric problems [26]. However, the number of controlled studies regarding the effectiveness of suicide survivor support groups is limited [27,28,29,30] and the more severe the grief process (irrespective of the cause of death), the more chance that therapeutic interventions will have positive results [31].

In addition, only a minority of survivors attend support groups and/or other services. A study by Provini *et al*. [23] showed that while 72% of survivors expressed the need for professional help, only 47% actually received it; an observation confirmed in other studies, e.g., [32]. It has been estimated that while there were over 50,000 suicides per year in Europe, only 10,000 survivors attended survivor groups and there are significant differences in the availability of this form of help in Europe. In general, the Western and Northern European countries offer more peer support opportunities than countries in Eastern and Southern Europe [33]. Besides the sheer availability of peer support, also other psycho-social factors determine how many survivors and who will use this type of support [10]. For some of the bereaved the informal help offered by family or friends is sufficient, others might fear admitting the need for help or try to avoid the stigma and negative social reactions related to suicide. Others, especially males, might reject any form of help or use other methods of coping, such as overworking or self-medication with alcohol or drugs. 

Suicide comprises a serious, although sometimes underestimated, occupational hazard for (mental) health professionals, and training and postvention for this group should be routinely available to reduce the psychological and public health cost of suicide. Mental health professionals as well as other professionals can be affected by suicide on both personal and professional levels [34,35]. A good professional training before the loss, help and support from the supervisor and colleagues after the loss and case reviews focused on learning (not blaming) can help the professional to deal with the suicide in an effective and constructive manner. On the other hand, legal or disciplinary proceedings, excessive media attention and professional isolation and stigmatization might lead to secondary traumatization and result in serious disruption of clinical practice [34,35].

### 4.2. Postvention from the Public Health Perspective

As Shneidman [7] has written, “a benign community ought routinely to provide immediate postventive mental health care for the survivor-victims of suicidal deaths” (p. 22). And indeed, during the past decades a number of countries, including US, UK, Ireland, New Zealand, Australia, Sweden, Norway, and the Flemish Region in Belgium, have developed comprehensive suicide prevention programs and policies which encompass postvention strategies. These include support-related activities (e.g., support groups, online resources, national suicide survivor days), awareness raising activities via dissemination of brochures, books as well as public walks and art exhibitions, and fundraising activities. For example, promotion of mental health of people bereaved by suicide is one of the goals of the National Suicide Prevention Strategy for England (Objective 2.8) [36]. Development of a support pack for survivors and people in contact with bereaved families (e.g., the police, religious leaders and general practitioners) is one of the initiatives undertaken to achieve this objective. The New Zealand Suicide Prevention Action Plan 2008–2012 includes development of policies, strategies and services in order to support families/whānau, friends and significant others bereaved by suicide, and culturally appropriate services for Māori (Goal 6) [37]. The Australian Living Is For Everyone (LIFE) Framework lists initiatives aimed at supporting individuals bereaved by suicide under Outcome 1.3 “Application and continued development of the evidence base for suicide prevention among high risk populations” and Outcome 5.3 “Reduced incidence of suicide and suicidal behaviour in the groups at highest risk” [38]. One of the projects funded by the strategy was the development of suicide bereavement support group standards in order to introduce best-practice guidelines and training programs for group facilitators [39]. The Flemish Suicide Prevention Action Plan consists of five clusters of actions, including development of programs for specific risk groups (Cluster 5) such as the people bereaved by suicide. Three new specific postvention targets were set: development of support and resources for suicide bereaved children and adolescents, implementation of professional standards for support groups, and implementation of media guidelines for reporting of suicide [40]. 

There remains of course the question if the social taboo relating to suicide and stigmatization of suicide survivors might not limit the effectiveness of such interventions and prevent some of the survivors in need from taking advantage of these resources. Grad [10] has proposed an interesting link between the magnitude of the problem of suicide in the society in general and the attitudes towards postvention. She suggested that “it would be interesting to know how the level of suicide in a society relates to the attitudes towards suicide survivors. Does the frequency of suicide help or hinder positive reactions towards suicide survivors?” ([10], p. 357). Unfortunately, to-date there have been no studies tackling this important public health issue. 

The evaluation of postvention programs is another important, albeit relatively neglected, public health challenge. A recent pioneering example of such a health-economic evaluation is the study of the Australian StandBy Response Service, a community-based outreach program providing support and a coordinated response for people bereaved through suicide [41]. The evaluation showed that the service appeared to reduce the negative impact of suicide bereavement on physical and mental health, including lower levels of suicidality, particularly within the first two years after the loss. In comparison to survivors who did not participate in the program, the StandBy clients reported higher levels of productivity both in terms of absenteeism and presenteeism (work attendance accompanied by low performance levels) and less frequent use of health care services, such as medical specialists and hospitals. Last but not least, the service saved society an average of AUD800 per person bereaved by suicide. Needless to say, such health-economic evaluations are much needed to provide evidence convincing private and public sponsors to further contribute to the building of “benign communities”. 

## 5. Postvention as Suicide Prevention and Directions for the Future

Suicide survivors comprise not only the target group of postvention activities; they are also an active force behind many suicide prevention initiatives, such as Suicide Prevention Action Network USA, Lifekeeper Memory Quilt in Australia and the Media Award for Responsible Portrayal of Suicide in Belgium. In the words of Paul Quinnett, “the suicide prevention movement has not been powered by rhetorical interest, but by the pain of those who have lost loved ones” (personal communication, AAS Suicidology List, 4 April 2011). Also, given the fact that suicide survivors are a group with increased suicide risk, postvention is an integral and indispensable component of comprehensive suicide prevention programs. Just as suicidology without the involvement of survivors would be poor suicidology, suicide prevention without survivors would be poor suicide prevention. 

The voice of survivors should be included in public health policies related to suicide prevention as well as involved in design and implementation of postvention programs and studies, although a detailed discussion of this issue is beyond the scope of this paper [17]. There is also a growing consensus in regards to the international policy and research agenda to ensure further progress in the field of postvention in the upcoming decades [2,42]. The major points include: (a) formulation of operational and consensus definitions of “a suicide survivor” and “postvention” and conducting further studies to establish the number of individuals affected by suicide (both survivors and people exposed to suicide), (b) conducting methodologically sound studies in order to identify the specific experiences and needs of various subgroups of survivors based upon age, gender, closeness and type of the relationship with the deceased, including cross-cultural research, and (c) conducting effectiveness studies of postvention activities, with a special focus on suicide support groups and public health policies, including health-economic studies. 
